# Włodzimierz Józef Godłowski (1900–1940)

**DOI:** 10.1007/s00415-023-11919-4

**Published:** 2023-08-05

**Authors:** Mateusz Mikołajczyk, Anna Pytasz-Kołodziejczyk, Anita Mikołajczyk

**Affiliations:** 1 Faculty of Dental Medicine, Medical University of Warsaw, Warsaw, Poland; 2 Institute of History, Warmia and Masuria University, Olsztyn, Poland; 3 School of Public Health, Warmia and Masuria University, Olsztyn, Poland

Włodzimierz Józef Godłowski (Fig. 1) was born on 7 November 1900 in Stryi (now in Ukraine) to Aleksander Godłowski, a physician, and Helena, née Bierzecka. In 1918, he passed his matriculation exam with honors in the State Gymnasium in Sanok, Poland, and was admitted to the Faculty of Medicine at the Jagiellonian University in Cracow. In November 1918, Godłowski volunteered for the Academic Battalion of the Polish Army and fought during the Polish–Ukrainian War. Godłowski remained in the reserve military force for less than a year, and in July 1920, he volunteered once again and took part in the Polish–Soviet War (1918–1921). He received a commemorative medal for that active service. Godłowski resumed his studies at Jagiellonian University in 1921. Between 1923 and 1924, he was employed as a student assistant in the Internal Medicine Clinic at St. Lazarus Hospital in Cracow under the supervision of Józef Latkowski [1].

**Fig. 1:**
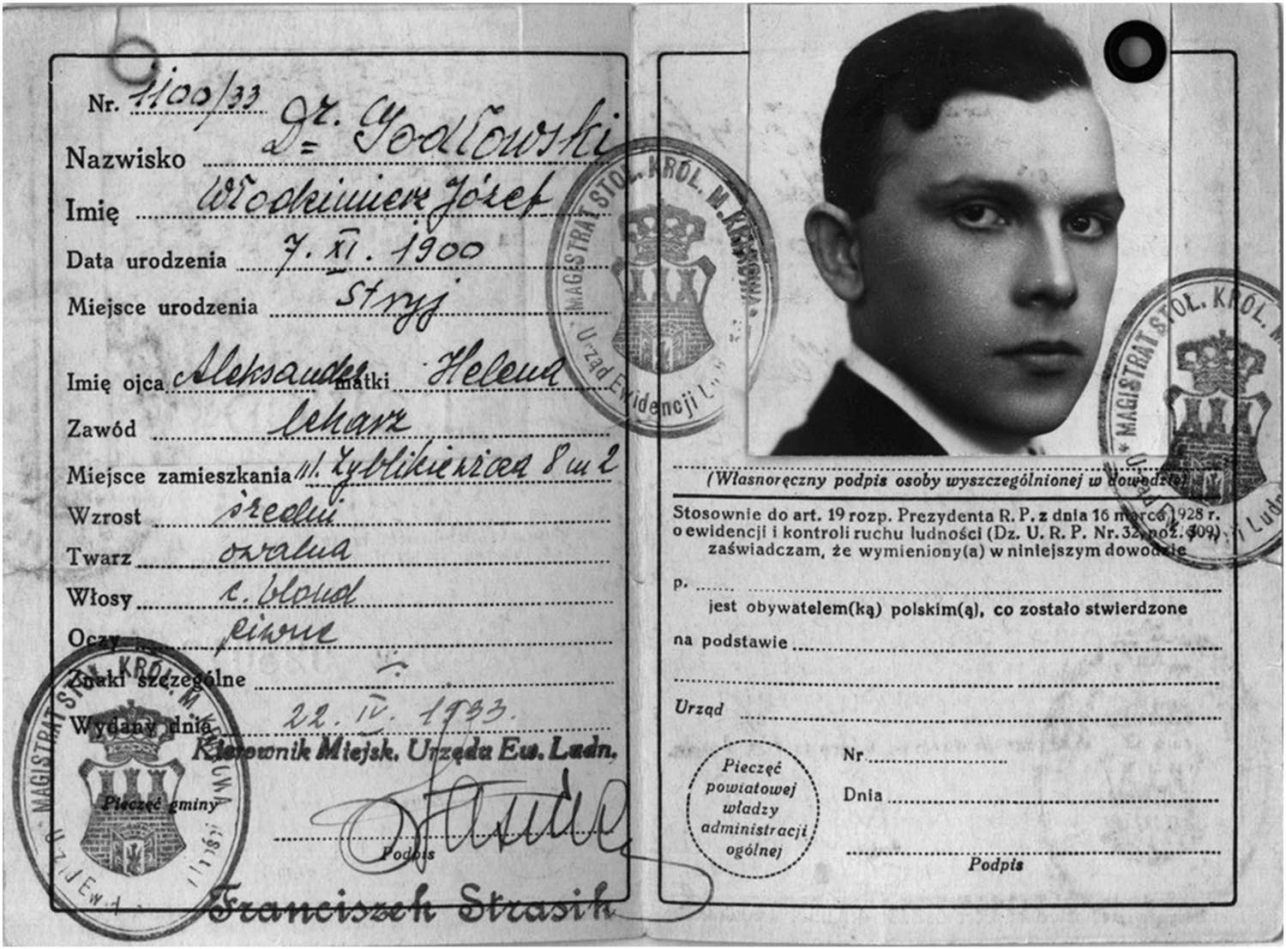
ID card of Włodzimierz Godłowski. Credit: From the family collection and the Katyn Institute, compiled by A. Rybicki. Accessed 25 May 2023 at: https://nasi-bliscy.federacja-katyn.org.pl/portrety/godlowski-wlodzimierz-jozef

Godłowski graduated in 1925, and became assistant at the Psychiatric Hospital in Rybnik, where he worked until September 1927, also conducting anatomical research. In November 1927, he was employed as assistant at the Neurological and Psychiatric Clinic of Jagiellonian University under the supervision of Jan Władysław Piltz [2]. Piltz created a clinical scholarship program for talented young physicians. Godłowski enrolled in the program and traveled to Vienna, where he worked with Otto Marburg at the Neurological Institute and Ernst Peter Pick at the Experimental Pharmacological Department for six months [3]. In 1930, Godłowski published the results of his research conducted in Vienna. His first research paper on the substantia nigra and Lewy bodies testified to his meticulous attention to details and his extensive knowledge of neuroanatomy. The article analyzed clinicopathologic correlations and detailed descriptions of methods for staining nigral cells [4]. In his article on diuresis, Godłowski provided evidence that pituitrin inhibits diuresis in decerebrated animals as well, which undermined the then erroneous views of Pick’s team [5].

Between 1931 and 1937, Godłowski was secretary of the Cracow Neurological Society [1]. He argued that neurological patients were admitted to hospital too late and most nasopharyngeal cancers were diagnosed at an advanced stage, which was why neurologists should make a rapid diagnosis. Godłowski reported on four initially misdiagnosed cases of nasopharyngeal cancer, and demonstrated neurological signs indicative of malignant tumors. He described the diagnosis, treatment, complications, and histopathology [6].

Godłowski conducted experiments on cats. He placed surgical lesions and identified nerve endings that received signals from taste bud cells in the pons and medulla. Moreover, he provided new descriptions of cranial nerves associated with taste (V, VII, and IX) [7]. He stimulated different parts of the brain stereotaxically. In November 1936, Godłowski defended his habilitation thesis [8]. The thesis summarized results of experiments on more than 300 animals, and constituted Godłowski’s most important scientific contribution. The author modified methods for stimulating subcortical areas and described structures involved in eye movement, with an emphasis on vestibular impulses and the thalamic reticular nucleus which receives input from sensory nuclei. He postulated that the results of his studies on eye movements and further research on vestibular pathways and saccadic eye movements were needed to improve diagnosis in otoneurology [8]. Godłowski’s research substantially contributed to clinical research on clinical neuroanatomy and neurophysiology. Godłowski observed that electrical stimulation of the thalamic internal medullary lamina (IML) in cats evoked eye movements. He recognized that electrical stimulation of the thalamic IML resulted in contralateral saccades, and suggested that the pulvinar-lateral posterior complex in the thalamus had an oculomotor function [8, 9].

Godłowski authored 15 scientific articles and monographs. Despite the fact that substantial progress has been made since, Godłowski’s discoveries had a lasting impact, as evaluations of ocular motor function are still a source of valuable diagnostic data. Godłowski was already a promising researcher in his early years, and his work received praise, as evidenced from correspondence with Otto Marburg and the Dutch neurologist Louis Jacob Joseph Muskens. In a letter to Godłowski dated 31 July 1936, Marburg wrote: “Your work has significant implications, although I do not agree on all points. It is quite possible that eye movement is coordinated by oculomotor nuclei, but in my opinion, the proposed system is too complex.” On 24 March 1937, Muskens wrote: “Your suggestion that specialized neural systems control saccadic eye movement is very interesting” [10].

In 1938, Godłowski was offered the position of head of the Department of Neurological and Mental Diseases at the Faculty of Medicine at Stefan Batory University, Vilnius (now in Lithuania) after the death of Maksymilian Rose. Godłowski accepted, but before assuming the new post, he was required to complete training at the Institute for Brain Research and General Biology in Neustadt in Schwarzwald, Germany, under Oskar Vogt. Godłowski worked there from August 1938 to April 1939 [1]. On 10 September 1938, Godłowski was appointed associate professor at the Faculty of Medicine of Stefan Batory University by the President of Poland [3].

After returning to Poland, Godłowski lectured and conducted research at Stefan Batory University [10]. He was scheduled to deliver an opening lecture at the beginning of the academic year on 1 October 1939. However, on 27 August 1939, Godłowski was conscripted into the Polish Army. After the outbreak of the Second World War, he was captured by the Soviet Army on 23 September 1939 and imprisoned in the Polish village of Radoszyn near Kowel (a region annexed by the USSR after the War, now part of Ukraine, since 1991). Godłowski was transported to a prisoner-of-war camp in Putyvl (USSR after the War, in Ukraine since 1991) and Kozelsk (USSR after the War, Russian Federation since 1991). He was one of the first Polish officers to be transported from Kozelsk to Katyn, where he was executed by the People’s Commissariat for Internal Affairs (NKVD) between 7 and 11 April 1940 by a shot in the head. Godłowski was survived by his widow Zofia, a laryngologist who never remarried, and their 5-year-old son Kazimierz, who became a renowned archeologist and served as a professor at Jagiellonian University [1, 3].

## Data Availability

Not applicable.
